# Comprehensive Analysis to Identify the Encoded Gens of Sodium Channels as a Prognostic Biomarker in Hepatocellular Carcinoma

**DOI:** 10.3389/fgene.2021.802067

**Published:** 2022-01-21

**Authors:** Yan Yan, Wen He, Yonghua Chen, Qiang Li, Jiahao Pan, Yunfei Yuan, Weian Zeng, Dongtai Chen, Wei Xing

**Affiliations:** ^1^ Department of Anesthesiology, Sun Yat-Sen University Cancer Center, State Key Laboratory of Oncology in South China, Collaborative Innovation Center for Cancer Medicine, Guangzhou, China; ^2^ Department of Anesthesiology, Huizhou Municipal Central Hospital, Huizhou, China; ^3^ Department of Anesthesiology, Peking University Shenzhen Hospital, Shenzhen, China; ^4^ Department of Hepatobiliary Oncology, Sun Yat-Sen University Cancer Center, State Key Laboratory of Oncology in South China, Collaborative Innovation Center for Cancer Medicine, Guangzhou, China

**Keywords:** SCN family, sodium channels, hepatocellular carcinoma, prognosis, Scn7a

## Abstract

The *SCN* family as the encoded gens of sodium channels has been proven to participate in development of cancers including hepatocellular carcinoma (HCC), but the prognostic value of the *SCN* family is unclear. The results of the UALCAN database had showed that *SCN2A/4A/5A/8A* mRNA were highly expressed in tumour tissues, while *SCN1A/7A/11A* mRNA were expressed at low levels (*p* < 0.05), furthermore, the expression of *SCN4A* and *SCN7A* had the similar levels in microarray analysis result. The pan-tumour analysis showed that *SCN7A* expression was stably lower in tumours than *SCN4A* expression by TIMER. Both *SCN4A* and *SCN7A* were related to tumour grade, nodal metastatic status, histological subtype, patient race, individual cancer stages and TP53 mutation status to varying degrees. The Kaplan–Meier plotter demonstrated that high *SCN4A* mRNA expression was correlated with better overall survival (OS), disease-specific survival (DSS) and progression-free survival (PFS) and that high expression of *SCN7A* mRNA was associated with better OS; however, in Asians, higher *SCN4A* was correlated with better OS and DSS, and higher *SCN7A* was well correlated with better OS, recurrence-free survival (RFS), DSS and PFS. Analysis of data from cBioPortal showed that mutation of *SCN7A* was related to RFS and PFS. The protein expression of *SCN4A* and *SCN7A* had been detected by Immunohistochemistry. Univariate survival analysis revealed that high *SCN7A* protein expression was significantly linked to better OS (*p* = 0.001) and RFS (*p* = 0.003). Moreover, *SCN7A* displayed as an independent prognostic factor by multivariate analysis. In addition, a lower methylation level indicated a poor outcome. Pathway and functional enrichment analysis predicted a relationship between *SCN7A* and the PI3K pathway. In conclusion, there are significant and stable changes in *SCN4A* and *SCN7A* expression in HCC. *SCN7A* expression has better prognostic value and might participate in HCC progression.

## Introduction

The current overall incidence and mortality of cancer are declining worldwide, but the incidence and mortality rates of liver cancer, one of the most common cancers in most countries, are on the rise (Bray et al., 2018). Hepatocellular carcinoma (HCC) accounts for the majority of adult liver cancer (Global Burden of Disease Liver Cancer et al., 2017). In China, HCC is the fourth most common cancer after lung, stomach and colorectal cancer, but it ranks second to lung cancer in terms of cancer-related death (Cao et al., 2020). There are many treatments for HCC, including surgical treatment, *trans*-arterial chemoembolization (TACE), and radiotherapy (Anwanwan et al., 2020). Among all therapeutic methods, surgical treatment is still the first-choice treatment for HCC; however, the surgical indications are limited. Therefore, finding a valuable biomarker to better evaluate the diagnosis and prognosis of HCC patients will be beneficial in the guidance of treatment and inhibition of metastasis.

At present, the most commonly used serum biomarker in the clinical diagnosis of HCC is *α*-fetoprotein (AFP); however, it has its own limitations, namely, that both its specificity and sensitivity are low. To improve the specificity and sensitivity of the HCC diagnosis, it is very important to study the mechanism of HCC occurrence, development and metastasis and to identify new biomarkers. Yunsheng Zhao (Zhao et al., 2017) found that in addition to classical biomarkers such as AFP and des-γ-carboxyprothrombin (DCP), newly discovered biomarkers for HCC include Golgi glycoprotein 73, glypican 3, transforming growth factor-β1, and insulin-like growth factor-II. However, it is still difficult to fully characterize HCC with a single biomarker. The molecular characteristics of HCC are not yet fully understood, and many new biomarkers still need to be discovered.

Sodium channel is a classical ion channel, and had been proved to provide vital effects in nervous system which played a key role in sensory transmission system (Bennett et al., 2019). According its special character, many clinical medicines aimed at sodium channel had been invent and used for managing pain, for example, local anesthetics, which had been widespread used in anesthesia and pain management for decades, even for now, they are still indispensable in anesthesia (Lirk et al., 2018). The encoded genes of sodium channel are the *SCN* family (*SCN1A-SCN11A*). *SCN* play a vital role in several pathophysiological processes, including those involved the physiological processes of tumours, such as colon, cervical, and prostate cancer, by manipulating cell proliferation, migration and invasion both *in vitro* and *in vivo* (House et al., 2010; Hernandez-Plata et al., 2012; Suy et al., 2012). However, the prognostic value of *SCN* family in HCC is still a mystery. In this research, we aimed to investigate the prognostic value of *SCN* family members and provide potential therapeutic targets for this challenging disease by using bioinformatic analysis.

## Materials and Methods

### Ethics Statement and Tissue Specimens

This study (Identification code, GZR 2017-130) was approved by the Clinical Research Ethics Committee of Sun Yat-sen University Cancer Center (Guangzhou, China), and it was conducted according to the principles expressed in the Declaration of Helsinki (Kilkenny et al., 2012). All data were obtained from online databases. Additionally, six pairs of HCC and corresponding non-tumour tissue samples and 306 tumour tissue samples were obtained from patients who had undergone hepatectomy in the Department of Hepatobiliary Oncology, Sun Yat-sen University Cancer Center, and informed consent was obtained from each patient. The patients were not treated with preoperative therapy and had no history of other malignancies. Patients with extrahepatic metastasis and HCC invading the biliary system were excluded (Yan et al., 2020). All the postoperative pathological results were hepatocellular carcinoma. The mode of recurrence were including intrahepatic and extrahepatic.

### UALCAN

UALCAN (http://ualcan.path.uab.edu/index.html) is a website for cancer data analysis based on The Cancer Genome Atlas (TCGA) database. It helps medical researchers analyze differences in gene transcription between tumours and normal samples and then perform more thorough analyses, such as identification and survival analysis of the biomarker, when they a target gene is found. Additionally, it can query related information in other databases through related links (Chandrashekar et al., 2017). In this study, the UALCAN technique was used to analyze the mRNA expression of the *SCN* family members and their relationships with clinicopathological parameters. The liver hepatocellular carcinoma of TCGA dataset was chosen, and the *SCN* family was scanned, then the results were shown.

### Microarray Analysis

Microarray analysis was performed as previously described (Yan et al., 2020). After purification and treatment according to the Agilent One-Colour Microarray-Based Gene Expression Analysis protocol (Agilent Technologies Inc., CA, United States), the tumour tissue was analyzed with Agilent Feature Extraction software (version 11.0.1.1, Agilent Technologies Inc.). In addition, the Genesspring GX V11.5.1 software package was used for quantile normalization and subsequent data processing. Screening conditions that were identified as statistically significant were a fold change (FC) ≥ 1.0 and a *p* value <0.05. A hierarchical clustering heatmap was drawn to visualize the difference between tumour and paired non-tumour tissues.

### TIMER

TIMER (http://timer.cistrome.org/) is a resource that includes many cancer types and is based on TCGA (Li et al., 2017). We performed a pan-tumor analysis of *SCN4A* and *SCN7A* through this website, and the data were compared with those from normal tissues. In the cancer exploration section, *SCN4A* and *SCN7A* were examined by Gene_DE procedure, then the results were shown.

### Kaplan-Meier Plotter

The Kaplan–Meier plotter is one of the largest data sets, containing 54,000 genes associated with survival in many types of cancer (Nagy et al., 2018). This study analyzed the association between the expression of *SCN4A* and *SCN7A* and overall survival (OS), recurrence-free survival (RFS), disease-specific survival (DSS), and progression-free survival (PFS) in liver cancer. Hazard ratios (HRs) with 95% confidence intervals (CIs) and *p* values are shown in each survival chart. Liver cancer dataset was chosen, The Draw Kaplan-Meier plot programs were performed to analyses the association between the expression of *SCN4A* and *SCN7A* and OS, RFS, DSS, and PFS respectively. Then the results were shown.

### cBioPortal

cBioPortal is an online and open resource tool that can be used to explore and analyze cancer genomic data (Gao et al., 2013). The connections between genetic mutations in *SCN4A* or *SCN7A* and OS, RFS, DSS or PFS in HCC were analyzed by using cBioPortal in this study. The liver hepatocellular carcinoma (TCGA, PanCancer Atlas) dataset was chosen, and the genomic profiles of mutations, putative copy-number alterations from GISTIC and mRNA expression were selected, the Patient/case Set was the samples with mRNA data. After entering the gene name of *SCN4A* and *SCN7A,* then the results were shown.

### Immunohistochemistry (IHC)

Immunohistochemistry (IHC) was performed as previously described (Chen et al., 2019). The HCC tissue slides rehydrated by means of an alcohol gradient and blocked with H_2_O_2_. Then, antigen retrieval was performed by microwave heating in citric acid buffer (pH 6.0). Next, tissues were incubated with primary antibodies at 4°C overnight. After incubation with the secondary antibody, the DAB chromogenic method was carried out for colorization. The slides were divided into four groups based on the principle described previously (Chen et al., 2019). The staining intensity were graded based on the follow standards: 0 = not stained, 1 = weakly stained, 2 = moderately stained, or 3 = strongly stained, and the percentage of positive tumor tissue were graded based on the follow standards: 0 (<10%), 1 (10–25%), 2 (26–50%), 3 (51–75%), or 4 (>75%). The two scores were multiplied, four categories of IHC staining were determined: absent staining (-) (score, 0–3), weak staining (+) (score, 4–6), moderate staining (++) (score, 7–9), and strong staining (+++) (score, 10–12).

### SurvivalMeth

SurvivalMeth is a web server used to investigate the effect of DNA methylation-related functional elements on prognosis based on data from TCGA, CCLE and GEO (Zhang et al., 2021). In this study, we analyzed the *SCN7A* DNA methylation level in HCC and its relationship with survival time as the outcome. In the Pre-Uploading section, single case procedure was carried out. After choosing the LIHC dataset and inputting the gene name, analysis could be done, the results were shown.

### GEPIA2

GEPIA2 is an updated and enhanced version of GEPIA that is used to provide more functionalities to support many kinds of analyses based on TCGA and GTEx data (Tang et al., 2019). We obtained 200 similar genes for comparisons with *SCN7A* in HCC tissue from this online tool. In the Similar Genes Detection section, LIHC dataset was selected, then the similar genes were obtained after running program.

### Metascape

Metascape is an online tool that can provide comprehensive analysis, including functional enrichment, interactome analysis, and gene annotation (Zhou et al., 2019). Functional enrichment was performed with 200 similar genes that were compared with *SCN7A* in GEPIA2. The gene list was pasted and the species of the genes were analyzed, then the functional enrichment could be performed.

### LinkedOmics

LinkedOmics is a free public database containing multiomics data for 32 cancer types, as well as mass spectrometry-based proteomics data and modifier data from the Clinical Proteomics Tumour Analysis Consortium (CPTAC) (Vasaikar et al., 2018). The LIHC cancer cohort was selected and the datatype was RNAseq. Then the hepatocellular carcinoma sample dataset was chosen, the target dataset was RNAseq data of LIHC. The statistical method was pearson correlation test. After finishing the procedure, pathway enrichment analysis in the KEGG pathway, Panther pathway and Wiki pathway databases could be performed by means of the LinkedOmics online tool.

### Statistical Analysis

Statistical analysis was performed as the previously described (Chen et al., 2017). All data were evaluated with GraphPad Software 6 (GraphPad, La Jolla, CA, United States) and Statistical Package for the Social Sciences (SPSS, version 22.0). The Kaplan-Meier method was used to evaluate the relationship between *SCN4A* or *SCN7A* expression and survival outcomes. The associations of clinicopathological parameters with the expression of *SCN4A* or *SCN7A* were verified by means of the chi-square test, and the Cox proportional hazards regression model was used to assess prognostic factors by univariate and multivariate analyses. A *p* value <0.05 indicated statistical significance.

## Result

### Different Expression of Different *SCN* Family Members in Patients With HCC

**FIGURE 1:**
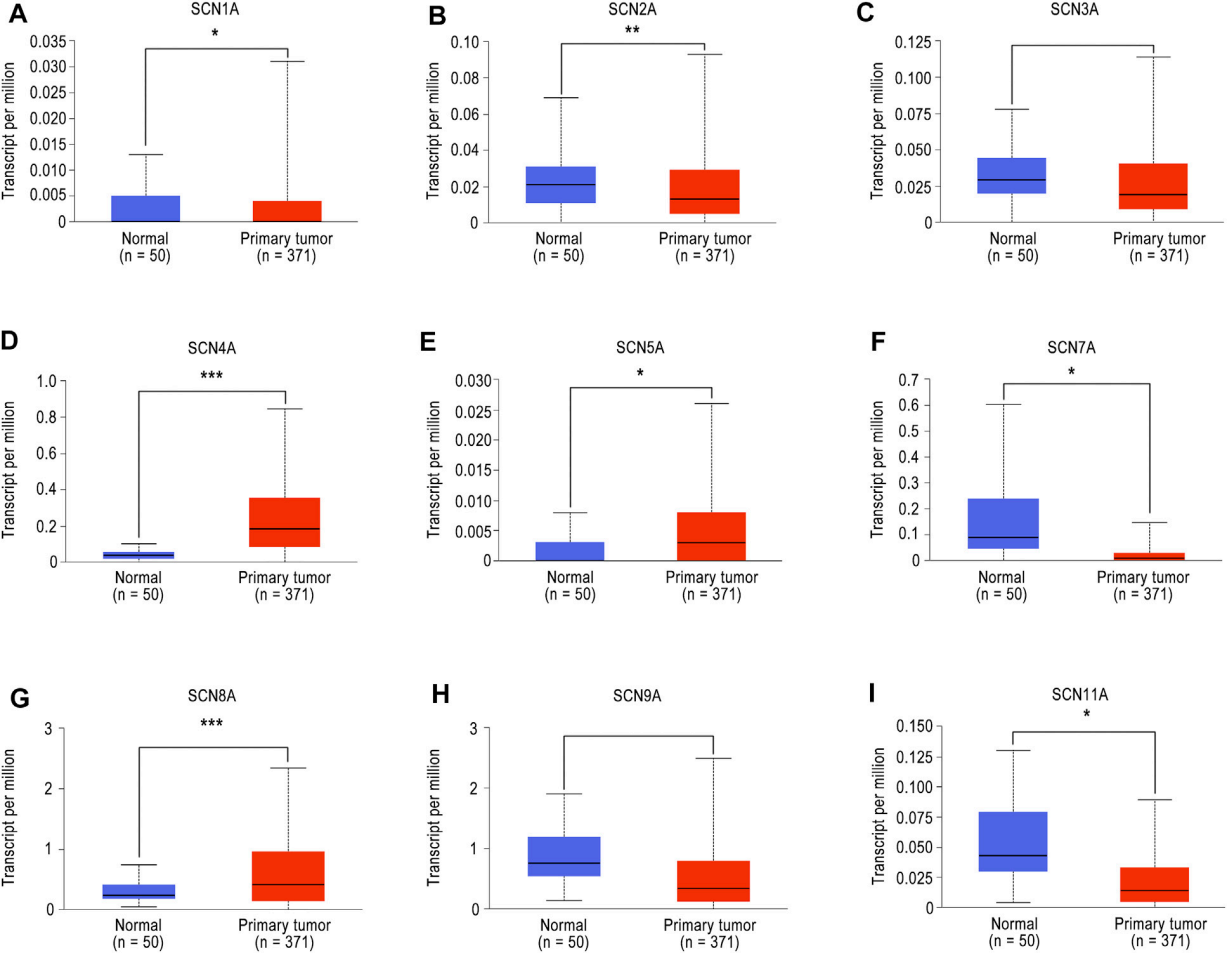
The mRNA expression of SCN family member in UALCAN. **(A)** *SCN1A* mRNA expression in tumour tissues and normal tissues. **(B)** *SCN2A* mRNA expression in tumour tissues and normal tissues. **(C)** *SCN3A* mRNA expression in tumour tissues and normal tissues. **(D)** *SCN4A* mRNA expression in tumour tissues and normal tissues. **(E)** *SCN5A* mRNA expression in tumour tissues and normal tissues. **(F)** *SCN7A* mRNA expression in tumour tissues and normal tissues. **(G)** *SCN8A* mRNA expression in tumour tissues and normal tissues. **(H)** *SCN9A* mRNA expression in tumour tissues and normal tissues. **(I)** *SCN11A* mRNA expression in tumour tissues and normal tissues. *p < 0.05, **p < 0.01, ***p < 0.001.

To investigate the expression of different *SCN* members in patients with HCC, we first used the UALCAN database to analyze the mRNA expression of different *SCN* members in normal tissues as well as primary tumours. As shown in Figure 1, we analyzed the expression levels of nine members of the *SCN* family. According to the results, *SCN4A/5A/8A* were highly expressed in tumour tissues, while *SCN1A/2A/7A/11A* were expressed at low levels (*p* < 0.05).

**FIGURE 2:**
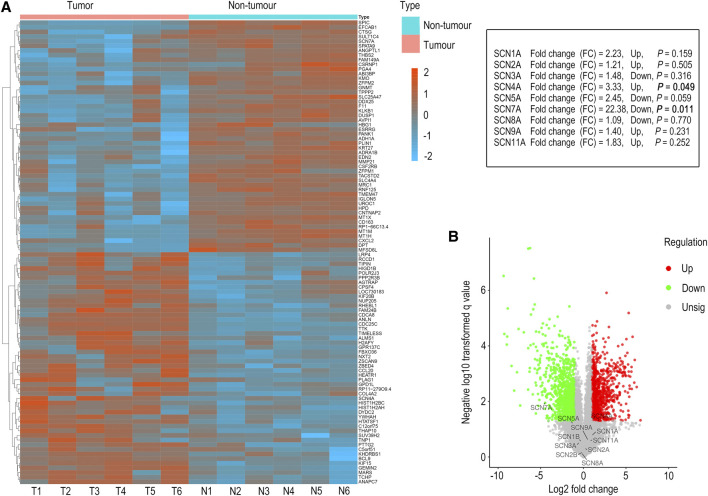
Microarray analysis of HCC tissues and adjacent non-tumour tissues. **(A)** The genes with significant changes in expression in HCC tissues and adjacent non-tumour tissues are shown in the hierarchical clustering heatmap. **(B)** All the genes in HCC tissues and adjacent non-tumour tissues are shown in the volcano plot.

To further verify the accuracy of information obtained from the UALCAN database, the expression of *SCN* family members in six pairs of tumour tissues and adjoined non-tumour tissues from HCC patients was examined by microarray analysis. As shown in Figure 2, only the expression of *SCN4A* (*p* = 0.049) and *SCN7A* (*p* = 0.011) was significantly changed; furthermore, the trends in *SCN4A* and *SCN7A* expression in tumour and non-tumour tissues were consistent with the results in the UALCAN database, in which the expression of *SCN4A* was higher in tumours and *SCN7A* was lower in tumours. Thus, *SCN4A* and *SCN7A* were the targets of future research.

### Pan-Tumor Analysis of *SCN4A* and *SCN7A*

Next, we performed a pan-tumour analysis of *SCN4A* and *SCN7A* in the TIMER database. The mRNA expression of *SCN4A* and *SCN7A* in 38 types of cancers was detected and compared with that in normal tissues. As shown in Supplementary Figure S1A, the expression of *SCN4A* was highly in several cancers, such as LIHC; however, its expression was lowly expressed in some other types of cancer tissues, such as BRCA and HNSC. The pan-tumour analysis of *SCN7A* is presented in Supplementary Figure S1B. From these results, we learned that expression of *SCN7A* was more stable and significantly lower in many types of non-cancer tissues compared to cancer tissues including HCC.

### Relationship Between *SCN4A/7A* and Clinicopathological Factors in Patients With HCC

Based on the UALCAN database, we analyzed the relationships between *SCN4A/7A* and pathological factors, including tumour grade, nodal metastatic status, histological subtype, patient race, individual cancer stages and TP53 mutation status. As shown in Supplementary Figure S1C, the mRNA expression of *SCN4A* was significantly higher at every grade, and the expression of *SCN7A* gradually decreased with the increase in tumour grade, but only significantly changed in grade 1 and grade 3. The mRNA expression level of *SCN4A/7A* was also significantly related to individual tumour stages; similarly, *SCN4A* was highly expressed in every stage, but *SCN7A* expression decreased in stage 3 and stage 4 (Supplementary Figure S1G). *SCN4A* was more highly expressed in tissues with nodal metastasis than in those without nodal metastasis. Although *SCN7A* expression was higher in the group without metastasis, the difference was not significant (Supplementary Figure S1D). In terms of histological subtypes, the expression of *SCN4A* and *SCN7A* was significantly different only in hepatobiliary carcinoma compared with that in normal tissues (Supplementary Figure S1E). From the results shown in Supplementary Figure S1F, we learned that only the cancer samples from Caucasian or Asian patients had higher *SCN4A* expression, while the cancer samples from African-American or Asian patients had lower *SCN7A* expression. The level of *SCN4A* expression was both higher in TP53 mutation and TP53 non-mutation groups, however, *SCN7A* expression only significantly decreased in tumour tissues with TP53 mutation (Supplementary Figure S1H).

### Survival Analysis of *SCN4A* and *SCN7A* in HCC by the Online Database

**FIGURE 3:**
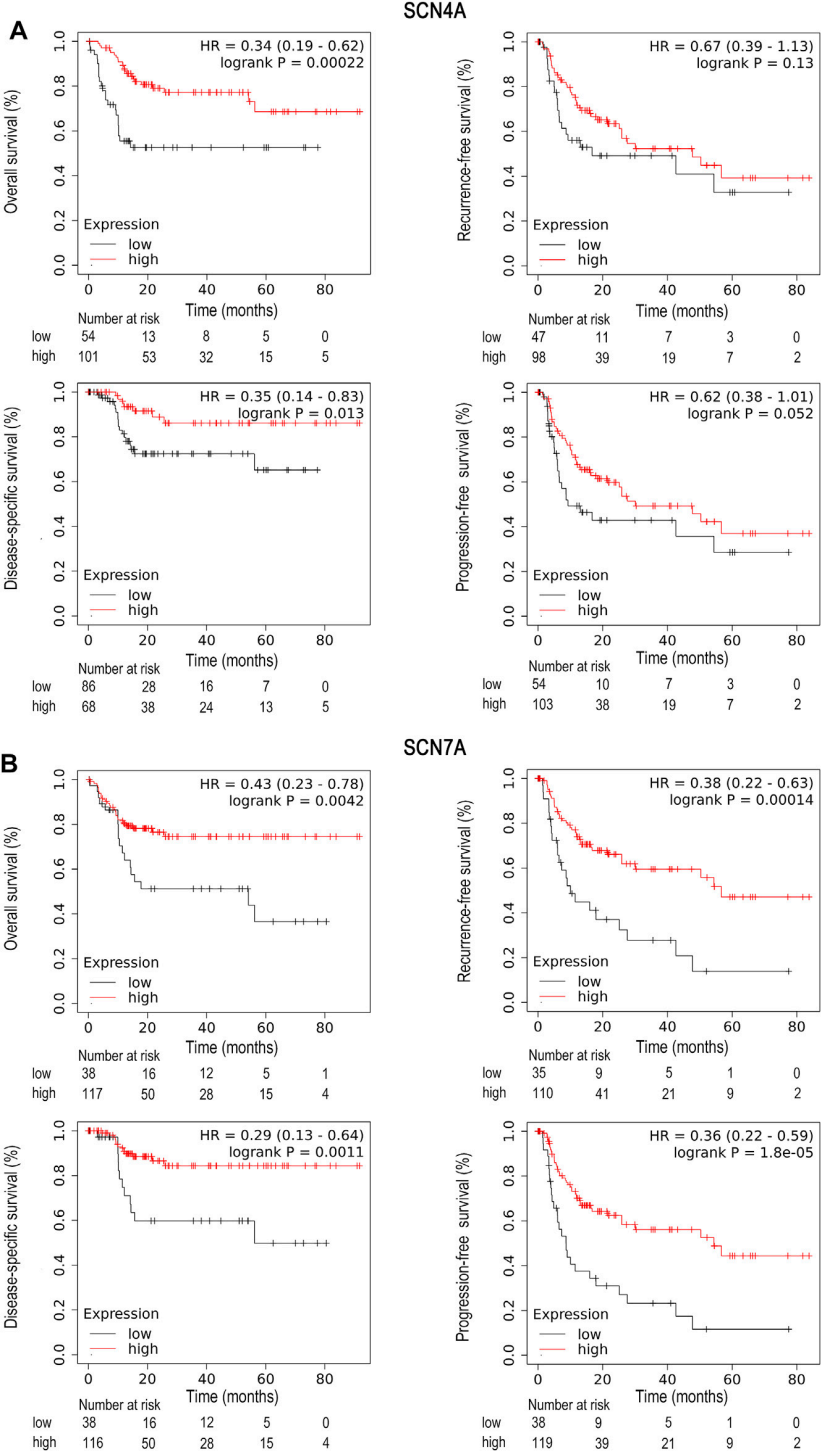
Survival analysis of SCN4A/7A mRNA in Asians of HCC by Kaplan–Meier plotter. **(A)** High *SCN4A* mRNA expression was correlated with better OS (*p* < 0.001) and DSS (*p* = 0.013). **(B)** High expression of *SCN7A* mRNA was well correlated with better OS (*p* = 0.004), RFS (*p* < 0.001), DSS (*p* = 0.001) and PFS (*p* < 0.001).

Survival analysis of *SCN4A* and *SCN7A* in HCC by means of the Kaplan–Meier plotter showed that high *SCN4A* mRNA expression was correlated with better OS (HR = 0.5, 95% CI: 0.35-0.72, and *p* < 0.001), DSS (HR = 0.55, 95% CI: 0.35-0.86, and *p* = 0.007) and PFS (HR = 0.67, 95% CI: 0.49-0.92, and *p* = 0.012) (Supplementary Figure S2A), while high expression of *SCN7A* mRNA was associated with better OS (HR = 0.63, 95% CI: 0.42-0.93, and *p* = 0.018) (Supplementary Figure S2B). In the previous analysis, we learned that the mRNA expression of *SCN4A* and *SCN7A* was significantly related to patient race. Given the situation of our hospital, where the majority of patients are Asian, other survival analyses included only Asians. In Asian patients, high expression of *SCN4A* mRNA was correlated with better OS (HR = 0.34, 95% CI: 0.19-0.62, and *p* < 0.001) and DSS (HR = 0.35, 95% CI: 0.14-0.83, and *p* = 0.013) (Figure 3A), and high expression of *SCN7A* mRNA was well correlated with better OS (HR = 0.43, 95% CI: 0.23-0.78, and *p* = 0.004), RFS (HR = 0.38, 95% CI: 0.22-0.63, and *p* < 0.001), DSS (HR = 0.29, 95% CI: 0.13-0.64, and *p* = 0.001) and PFS (HR = 0.36, 95% CI: 0.22-0.59, and *p* < 0.001) (Figure 3B).

**FIGURE 4:**
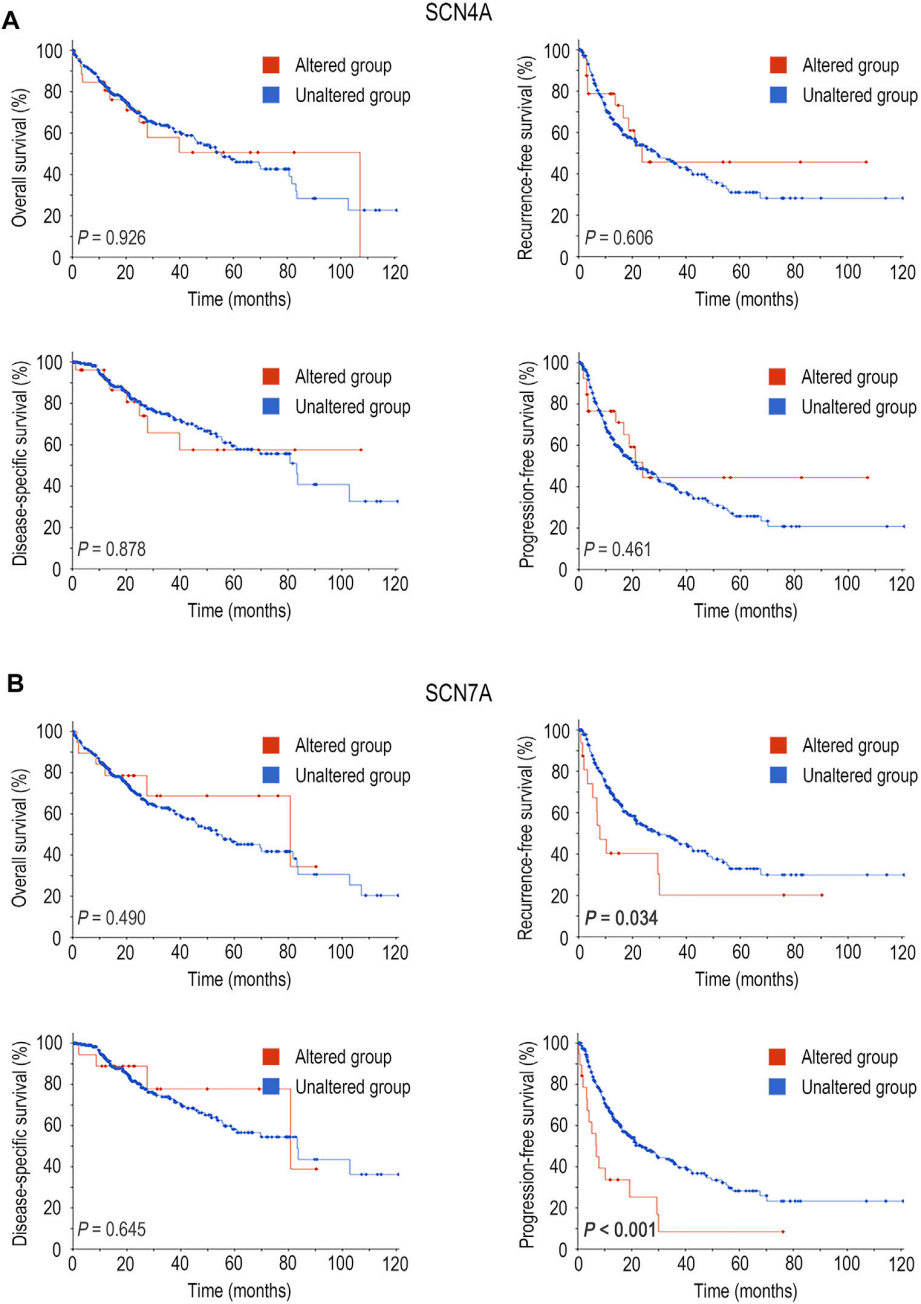
Survival analysis of SCN4A/7A mutations in HCC by cBioPortal. **(A)** *SCN4A* mutation was not related to OS (*p* > 0.05), RFS (*p* > 0.05), DSS (*p* > 0.05) or PFS (*p* > 0.05). **(B)** *SCN7A* alteration was related to RFS (*p* = 0.034) and PFS (*p* < 0.001).

Furthermore, the relationship between mutations in *SCN4A* and *SCN7A* and the survival of patients with HCC was examined by means of the cBioPortal database. *SCN4A* mutation was not related to OS (*p* > 0.05), RFS (*p* > 0.05), DSS (*p* > 0.05) or PFS (*p* > 0.05) (Figure 4A), but *SCN7A* alteration was related to RFS (*p* = 0.034) and PFS (*p* < 0.001) (Figure 4B).

### The Prognostic Value and Expression of *SCN4A* and *SCN7A* Protein in HCC Tissues

**FIGURE 5:**
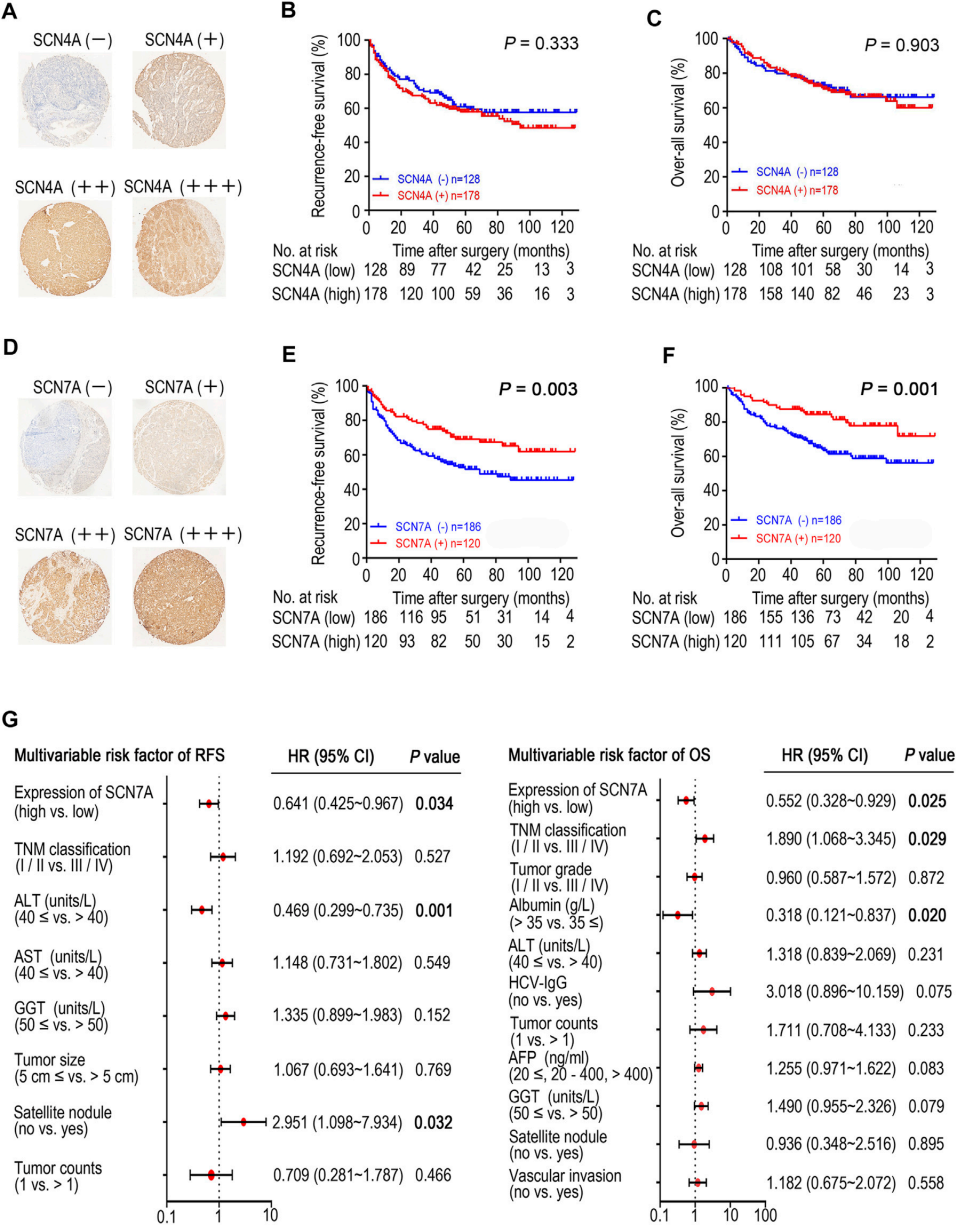
The expression of SCN4A/7A protein in HCC by immunohistochemistry and the survival analysis of patients with SCN4A/7A protein expression. **(A)** *SCN4A* protein staining in HCC. **(B)** *SCN4A* protein expression was not related to RFS (*p* > 0.05). **(C)** *SCN4A* protein expression was not related to OS (*p* > 0.05). **(D)** *SCN7A* protein staining in HCC. **(E)** Higher expression of *SCN7A* protein was related to longer RFS time (*p* = 0.003). **(F)** Higher expression of *SCN7A* protein was related to longer OS time (*p* = 0.001). **(G)** Cox multivariate analysis of contributory factors to recurrence-free survival and overall survival.

Based on the IHC results, four different staining intensities were grouped (Figures 5A,D). A total of 306 patients with a mean age of 50.7 (range 20–83) years comprised 270 males and 36 females whose median follow-up period was 59.1 (range 1–129) months. As shown in Figures 5B,C, *SCN4A* protein expression was not connected to OS or RFS (*p* > 0.05); however, *SCN7A* protein expression was significantly linked to OS (*p* = 0.001) and RFS (*p* = 0.003) (Figures 5E,F), and higher expression was associated with better survival, which was consistent with the results of the online databases. In addition, *SCN7A* expression was related to patient age (*p* < 0.001), satellite nodules (*p* = 0.028), tumour size (*p* = 0.001), adjacent organ invasion (*p* = 0.021), ICGI15 (*p* = 0.043), tumour grade (*p* = 0.034) and TNM classification (*p* < 0.001) (Supplementary Table S2). Univariate survival analysis revealed that AFP (*p* = 0.038), GGT (*p* = 0.003), satellite nodules (*p* < 0.001), vascular invasion (*p* = 0.004), tumour counts (*p* < 0.001), HCV-IgG (*p* = 0.022), ALT (*p* = 0.007), albumin (*p* = 0.045), tumour grade (*p* = 0.005) and TNM classification (*p* < 0.001) were related to OS, while GGT (*p* = 0.002), tumour size (*p* = 0.009), satellite nodules (*p* < 0.001), tumour counts (*p* = 0.002), AST (*p* = 0.004), ALT (*p* = 0.004) and TNM classification (*p* < 0.001) were correlated with RFS (Supplementary Table S1). Multivariate analysis showed that *SCN7A* (*p* = 0.034), satellite nodules (*p* = 0.032) and ALT (*p* = 0.001) were independent prognostic factors for RFS. Furthermore, *SCN7A* (*p* = 0.025), as well as albumin (*p* = 0.020) and TNM classification (*p* = 0.029), was also an independent prognostic factor for OS (Figure 5G and Supplementary Table S3).

### The DNA Methylation Level of *SCN7A* and Its Prognostic Value in HCC

**FIGURE 6:**
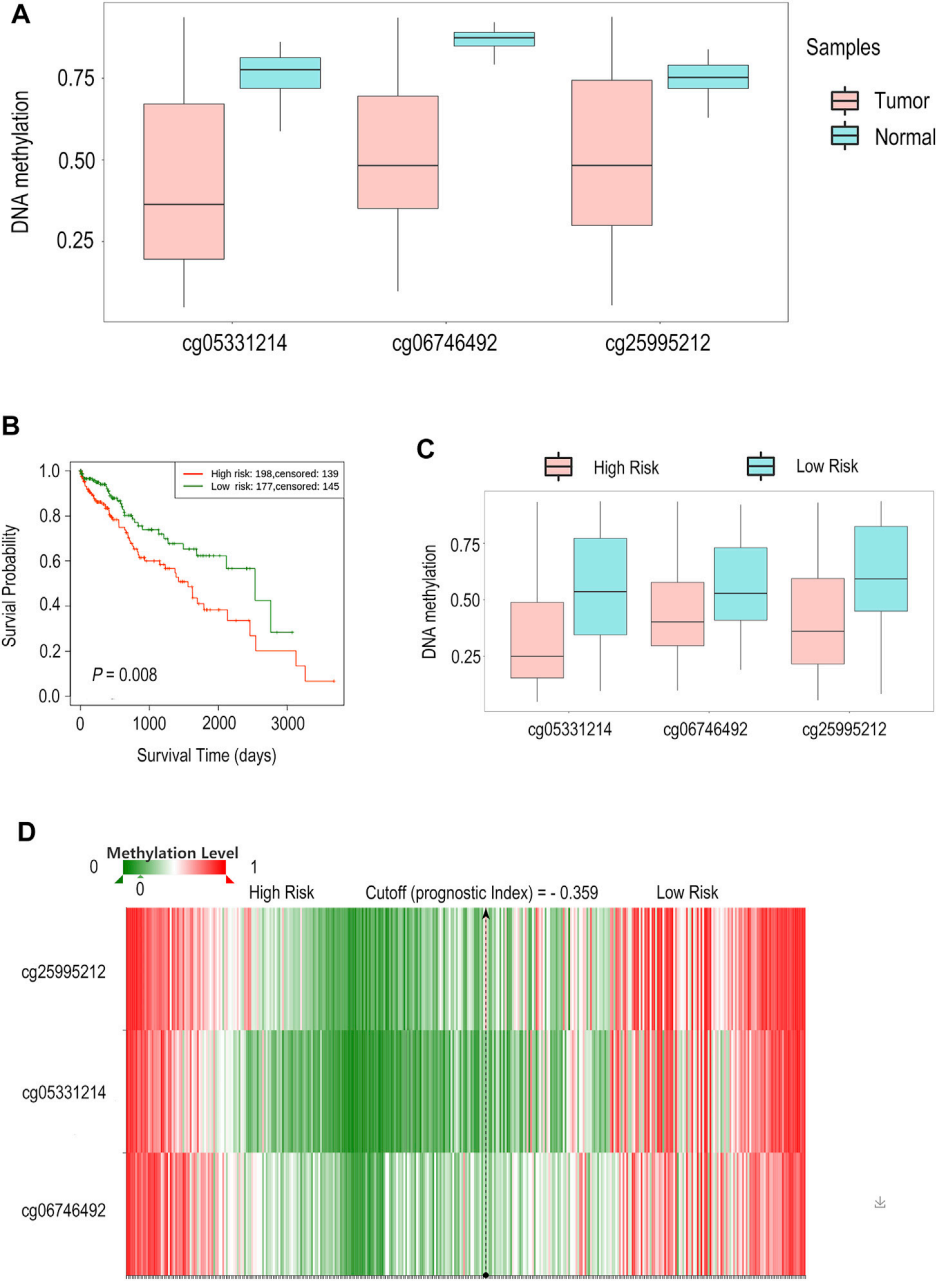
The DNA methylation level of SCN7A and its prognostic value in HCC by SurvivalMeth. **(A)** A lower *SCN7A* DNA methylation level with all probes was shown in HCC tumour tissues. **(B)** The high-risk group of HCC patients had poor outcomes. **(C)** The High-risk group had lower methylation levels of CpGs. **(D)** The methylation level of CpGs is shown in the hierarchical clustering heatmap.

According to previous results, we found that both *SCN7A* mRNA and protein expression were related to the outcome of HCC patients. Upon further exploration, a lower *SCN7A* DNA methylation level was shown with all probes in HCC tumour tissues (Figure 6A) based on the results of the SurvivalMeth online tool. The high-risk group, which had a poor outcome among HCC patients (Figure 6B), had a lower methylation level of CpGs (Figures 6C,D). As shown above, the DNA methylation level of *SCN7A* was low in tumours, and the lower methylation level might indicate a poor outcome.

### Predicted Pathway and Functions Enrichment Analysis of *SCN7A* and 200 Most Frequently Altered Neighboring Genes in HCC

Pathway enrichment analysis of *SCN7A* in the KEGG pathway, Panther pathway and Wiki pathway databases was performed by the LinkedOmics online tool, and the results are shown in Supplementary Figure S3A–C. The 200 most frequently altered neighbouring genes were found by GEPIA2 (Supplementary Table S4). The PI3K pathway, which was one of the most likely pathways to be related to the progression of HCC, was found in the results of the pathway and function enrichment analysis of *SCN7A* and the 200 most frequently altered neighboring genes (Supplementary Figure S3D,E), and the PI3K pathway was also found by LinkedOmics.

## Discussion

HCC is one of the most common cancers in the world, and the mortality rate has been on the rise in recent years. Therefore, it is of great significance to study the pathogenesis of HCC and find biomarkers for disease diagnosis. Many studies have demonstrated that lidocaine has an important anti-tumour function because it can indirectly or directly affect the biological characteristics of cancer cells; for example, it can suppress cell proliferation, induce cell apoptosis, and inhibit cell migration (Sakaguchi et al., 2006; Chang et al., 2014a; Chang et al., 2014b; Piegeler et al., 2015). *SCNs* have been reported in a variety of cancers, such as breast cancer, colon cancer, and lung cancer, but they are still rarely studied in liver cancer. Therefore, this study aimed to analyze the expression of *SCN* family members in HCC and determine their prognostic value to improve the accuracy of prognosis in HCC patients.

The mRNA levels of different *SCN* family members are different in HCC in the UALCAN database; however, the mRNA levels of *SCN4A* and *SCN7A* were the same in the microarray analysis, indicating that these subtypes were stably expressed in HCC. Additionally, the expression of *SCN4A* was higher in tumours, while that of *SCN7A* was lower, suggesting that *SCN4A* and *SCN7A* might have different effects in HCC. Furthermore, the expression of *SCN7A* was steady in most tumours, similar to glycosylation markers, which have already been used as cancer markers in the clinic (Silsirivanit, 2019). The only difference was that the levels of glycosylation markers were higher in tumours, while that of *SCN7A* was lower.

Overall, the survival analyses in this study showed that *SCN7A* had better prognostic value in Asian patients with HCC. Higher *SCN7A* expression regardless of mRNA or protein expression was related to a better outcome in HCC patients, and even the DNA mutation and DNA methylation level of *SCN7A* could distinguish the different outcomes in HCC. However, the Kaplan–Meier plotter analysis showed that *SCN4A* had better prognostic value when the subjects were not restricted to Asian patients. All the tissues for the IHC experiments were from Asians, which was a limitation of this study, so the prognostic value of *SCN4A* might be underestimated here. During this study, we made an interesting observation that the DNA methylation level of *SCN7A* was lower in tumours and that the mRNA expression of *SCN7A* was also lower in tumours. In most cases, a lower DNA methylation level means a higher mRNA level. However, in recent years, an increasing number of studies have found that higher DNA methylation levels lead to higher mRNA levels in cancer cases (Castelo-Branco et al., 2013; Lee et al., 2018; Takasawa et al., 2018; Rodger et al., 2019; Smith et al., 2020). Therefore, the DNA methylation level and the mRNA expression of *SCN7A* could both be lower in tumours, and this result is consistent with the literature.

The results of pathway enrichment analysis showed that the PI3K-Akt pathway was related to *SCN7A*. The PI3K-Akt pathway has been proven both *in vitro* and *in vivo* to participate in the progression of many types of cancer (Chamcheu et al., 2019; Ediriweera et al., 2019; Lee et al., 2020). Previous studies have already found that high expression of *SCN8A* could enhance the invasion of cervical cancer cells (Hernandez-Plata et al., 2012) and that *SCN9A* could promote gastric cancer progression (Xia et al., 2016). The alterations in *SCN7A* expression in HCC might not just be a simple phenomenon; *SCN7A* might be involved in the progression of HCC.

In this study, we analyzed the expression of all members of the *SCN* family in HCC and evaluated the prognostic value of *SCN4A* and *SCN7A*. Higher *SCN7A* expression predicted a better outcome for HCC patients, and *SCN7A* might take part in the progression of HCC. To further characterize the role *SCN7A* plays in HCC, more studies need to be designed and implemented.

## Data Availability

The datasets presented in this study can be found in online repositories. The names of the repository/repositories and accession number(s) can be found below: https://www.researchdata.org.cn/default.aspx, RDDB2021520042.

## Supplementary Material

Supplementary Figure S1 Pan-tumour analysis of SCN4A/7A and the relationship between SCN4A/7A and clinicopathological factors in patients with HCC. **(A)** The pan-tumour analysis of *SCN4A*. **(B)** The pan-tumour analysis of *SCN7A*. **(C)** The relationship between *SCN4A/7A* and tumour grade. **(D)** The relationship between *SCN4A/7A* and nodal metastatic status. **(E)** The relationship between *SCN4A/7A* and histological subtype. **(F)** The relationship between *SCN4A/7A* and patient race. **(G)** The relationship between *SCN4A/7A* and individual cancer stages. **(H)** The relationship between *SCN4A/7A* and TP53 mutation status. * *P* < 0.05, ** *P* < 0.01, *** *P* < 0.001.

Supplementary Figure S2 Survival analysis of SCN4A/7A mRNA in HCC by Kaplan–Meier plotter. **(A)** High *SCN4A* mRNA expression was correlated with better overall survival (OS) (*P* < 0.001), disease-specific survival (DSS) (*P* = 0.007) and progression-free survival (PFS) (*P* = 0.012). **(B)** High *SCN7A* mRNA expression was correlated with better overall survival (OS) (*P* = 0.018).

Supplementary Figure S3 Pathway enrichment analysis of SCN7A by LinkedOmics and Metascape. **(A)** Pathway enrichment analysis of KEGG pathways by LinkedOmics. **(B)** Pathway enrichment analysis of the Panther pathway by LinkedOmics. **(C)** Pathway enrichment analysis of the Wiki pathway by LinkedOmics. **(D)** Pathway and function enrichment analysis of *SCN7A* and 200 most frequently altered neighbouring genes in HCC by Metascape. **(E)** Network of enriched terms in pathway and functional enrichment analysis by Metascape.

## Abbreviations

VGSC, voltage-gated sodium channel; HCC, hepatocellular carcinoma; OS, overall survival; RFS, recurrence-free survival; DSS, disease-specific survival; PFS, progression-free survival; PI3K, phosphatidylinositol-3-kinase.
