# Supplementary figures and images for: Comprehensive Analysis to Identify the Encoded Gens of Sodium Channels as a Prognostic Biomarker in Hepatocellular Carcinoma
Source: Front Genet. 2022 Jan 21;12:802067. doi: 10.3389/fgene.2021.802067 (PMC8815461; doi:10.3389/fgene.2021.802067)

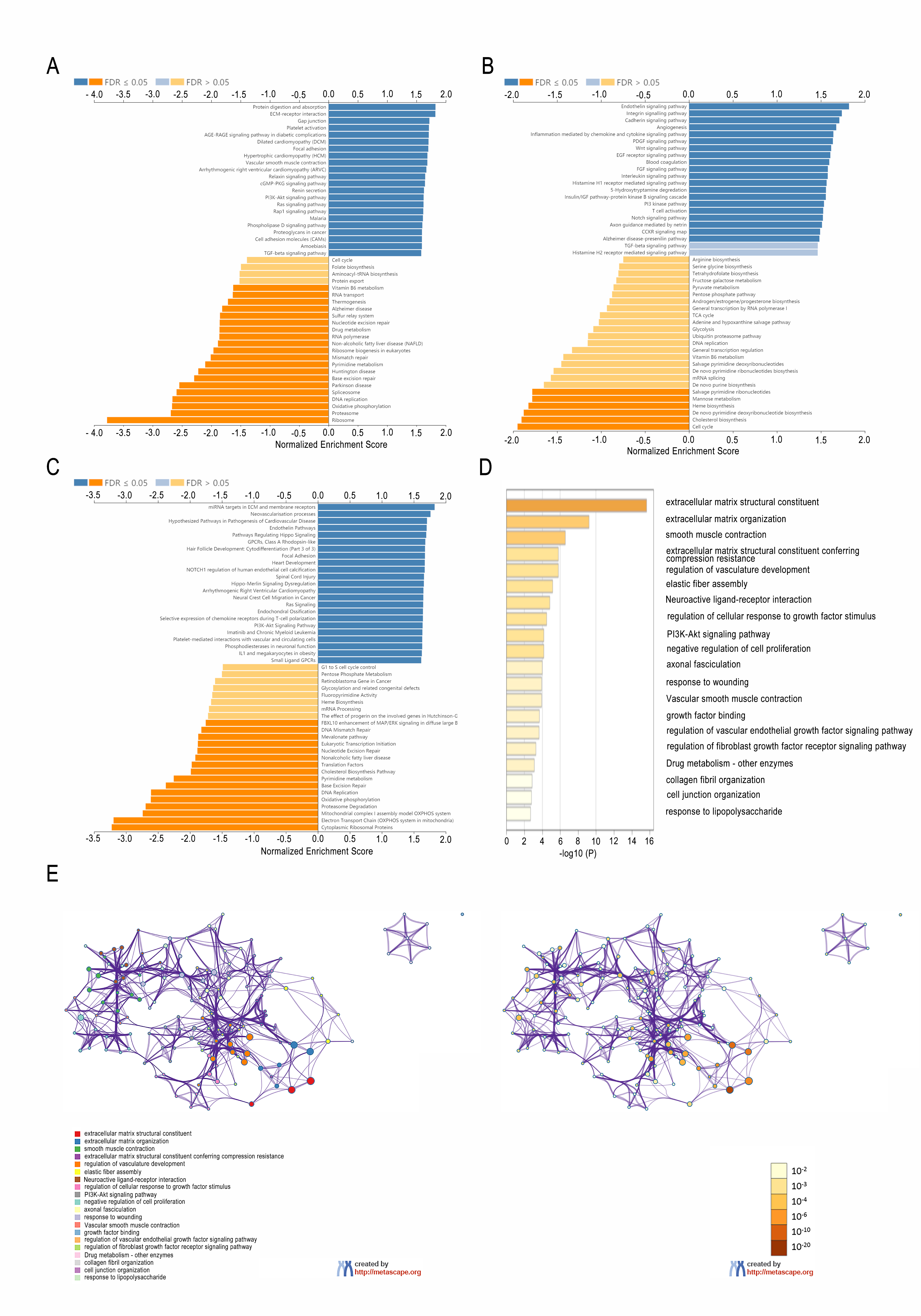

Supplement: Supplementary file 1 [file Image3.jpeg]

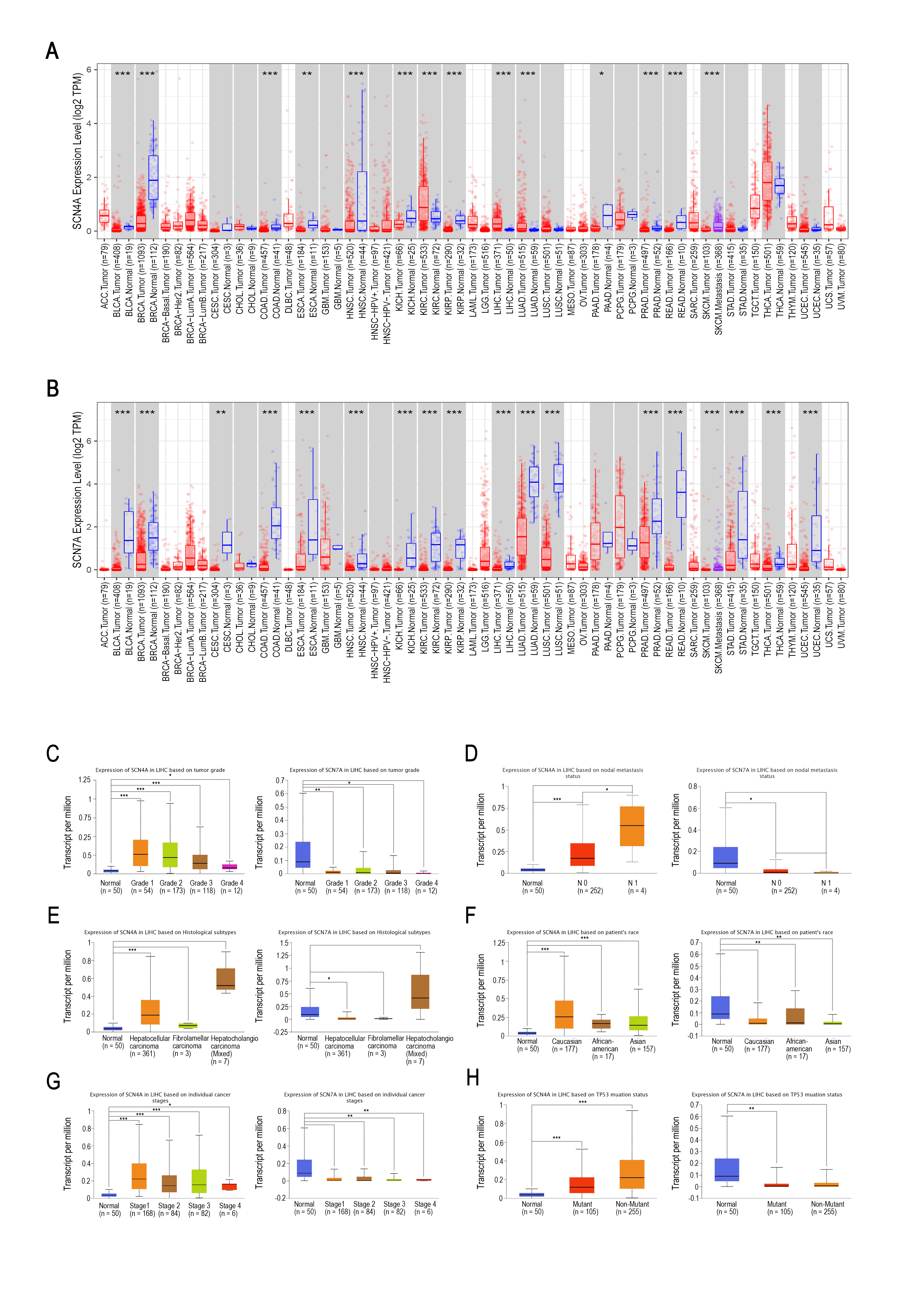

Supplement: Supplementary file 2 [file Image1.jpeg]

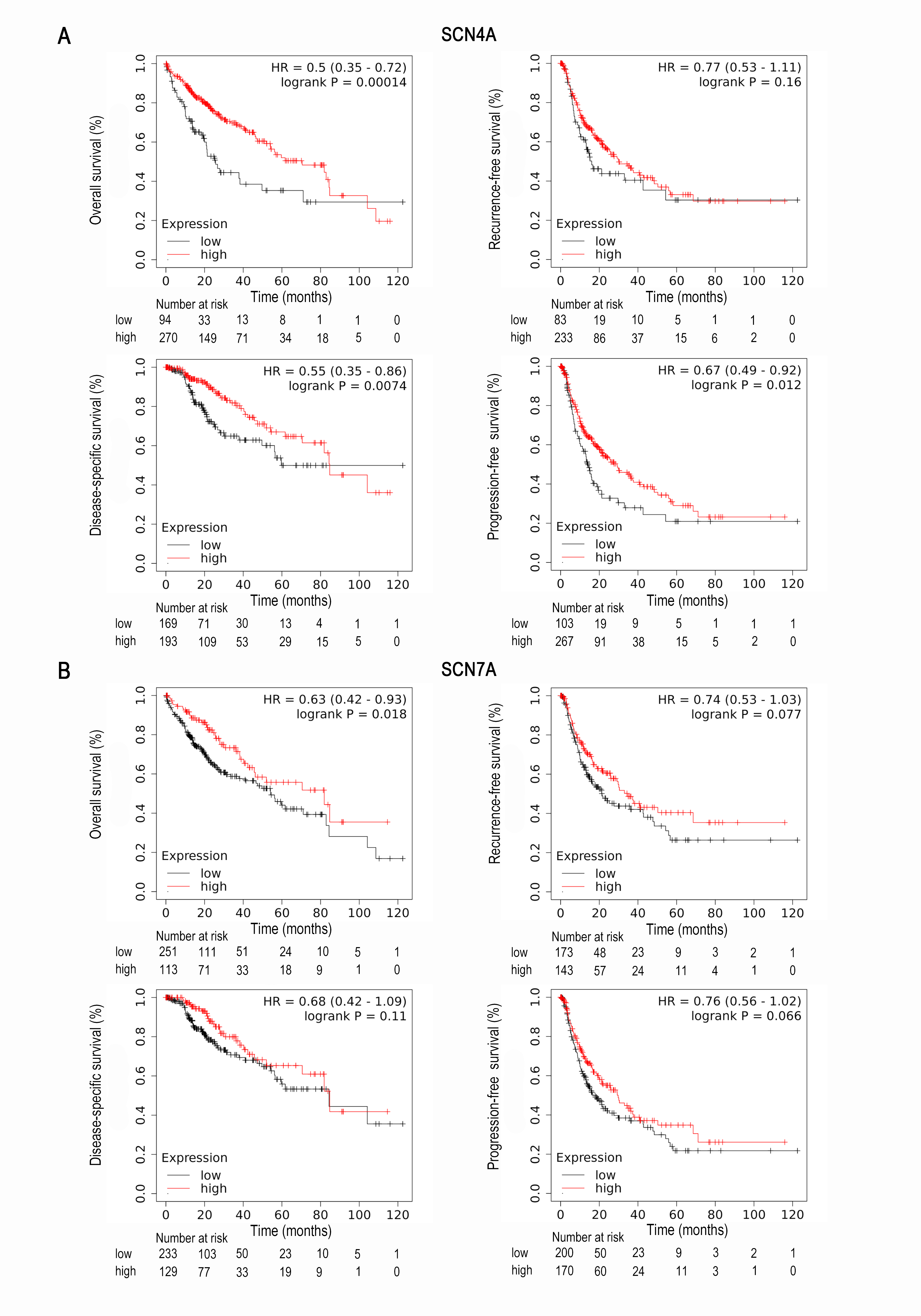

Supplement: Supplementary file 3 [file Image2.jpeg]
