# Supplementary material for: Comprehensive Analysis to Identify the Encoded Gens of Sodium Channels as a Prognostic Biomarker in Hepatocellular Carcinoma
Source: Front Genet. 2022 Jan 21;12:802067. doi: 10.3389/fgene.2021.802067 (PMC8815461; doi:10.3389/fgene.2021.802067)
Supplement: Supplementary file 4 [file DataSheet2.doc]

| Gene Symbol | PCC | Gene Symbol | PCC | Gene Symbol | PCC | Gene Symbol | PCC | Gene Symbol | PCC | Gene Symbol | PCC | Gene Symbol | PCC | Gene Symbol | PCC |
| --- | --- | --- | --- | --- | --- | --- | --- | --- | --- | --- | --- | --- | --- | --- | --- |
| C7 | 0.62 | RP4-761I2.5 | 0.58 | ZFPM2 | 0.56 | RP11-142G1.3 | 0.53 | LTBP2 | 0.51 | NFKBIA | 0.49 | FBN1 | 0.47 | KRTAP19-7 | 0.46 |
| LINC00092 | 0.62 | OR2J1 | 0.58 | KRTAP22-2 | 0.56 | TRAV1-2 | 0.53 | PABPC5 | 0.51 | FAM74A3 | 0.49 | RN7SKP76 | 0.47 | RP11-35L17.3 | 0.46 |
| OMD | 0.62 | RNU1-108P | 0.58 | RP11-126O22.6 | 0.56 | RP11-433P17.3 | 0.53 | RP11-445P19.3 | 0.51 | LINC00919 | 0.49 | PRKG1 | 0.47 | GABRG1 | 0.46 |
| CRISPLD2 | 0.61 | RP11-471G13.2 | 0.58 | FBLN5 | 0.56 | CTD-2033D15.3 | 0.53 | TNXB | 0.51 | RPL10P1 | 0.49 | AMY1C | 0.47 | AL163953.3 | 0.46 |
| LINC00989 | 0.6 | RP11-393N4.1 | 0.58 | TSKS | 0.55 | RP11-81F13.1 | 0.53 | SLC17A6 | 0.51 | AP000641.1 | 0.49 | CACNA1C | 0.47 | ZNF849P | 0.46 |
| SVEP1 | 0.6 | OR2P1P | 0.58 | AP000470.2 | 0.55 | MMRN1 | 0.53 | RP11-395E19.4 | 0.51 | TEK | 0.49 | RP11-433P17.2 | 0.47 | OR2B3 | 0.46 |
| TRPC2 | 0.6 | PGAM4P2 | 0.58 | AC008565.1 | 0.55 | LINC01036 | 0.53 | C7orf66 | 0.51 | MAGEC3 | 0.49 | GEM | 0.47 | RPS23P2 | 0.46 |
| EFS | 0.59 | MTND3P1 | 0.58 | ITGA9 | 0.55 | RP11-45F15.2 | 0.52 | RP11-126O22.7 | 0.5 | CTD-2533K21.2 | 0.48 | RP11-216P16.4 | 0.47 | RP11-274H24.1 | 0.46 |
| LAMA2 | 0.59 | ZNF385D-AS2 | 0.58 | XXbac-BPG258E24.10 | 0.55 | RP11-164J13.1 | 0.52 | MYOCD | 0.5 | CTD-2647E9.1 | 0.48 | PEAR1 | 0.47 | EFEMP1 | 0.46 |
| UGT2A2 | 0.58 | RP11-466F5.10 | 0.58 | RP11-347H15.2 | 0.55 | SRL | 0.52 | CTC-546K23.2 | 0.5 | INMT | 0.48 | ADAMTS1 | 0.47 | GHSR | 0.46 |
| OR8X1P | 0.58 | RP11-731N10.1 | 0.58 | COL14A1 | 0.55 | CSNK1A1P1 | 0.52 | RP11-1H8.5 | 0.5 | EPHA3 | 0.48 | ADAMTS9-AS1 | 0.47 | RP3-400B16.2 | 0.46 |
| AC073387.2 | 0.58 | SDHDP1 | 0.58 | RNF180 | 0.55 | RP11-81K2.2 | 0.52 | ADORA3 | 0.5 | RP11-168O22.1 | 0.48 | RP11-549A6.1 | 0.47 | RP11-488L18.4 | 0.45 |
| RP11-410B16.1 | 0.58 | RP11-466F5.6 | 0.58 | GALNT16 | 0.55 | USP32P3 | 0.52 | RP11-815J21.4 | 0.5 | THBS2 | 0.48 | AC000403.4 | 0.47 | PPP1R12B | 0.45 |
| AC013448.2 | 0.58 | ITGBL1 | 0.58 | RP11-126O22.3 | 0.55 | SYNPO2 | 0.52 | RP11-673E1.3 | 0.5 | DACT1 | 0.48 | UQCRBP2 | 0.47 | RP11-314D7.3 | 0.45 |
| RP11-353J17.3 | 0.58 | RGS7BP | 0.58 | DCN | 0.55 | ST8SIA6 | 0.52 | RP11-231E6.1 | 0.5 | LINC01197 | 0.48 | CCDC144CP | 0.47 | F2R | 0.45 |
| PRPS1P1 | 0.58 | DUXA | 0.58 | RN7SL89P | 0.54 | DPT | 0.52 | FXYD6 | 0.5 | SVOP | 0.48 | ZKSCAN7P1 | 0.46 | MYH11 | 0.45 |
| RP11-289E15.1 | 0.58 | RP11-815J4.5 | 0.57 | OGN | 0.54 | SUMO4 | 0.52 | AC026150.6 | 0.5 | RP11-134O21.1 | 0.48 | CPXM2 | 0.46 | NMUR1 | 0.45 |
| RP11-450H5.4 | 0.58 | UGT2A1 | 0.57 | SAR1P1 | 0.54 | RP11-672A2.4 | 0.52 | NCAM2 | 0.5 | ASPN | 0.48 | CTD-2140G10.2 | 0.46 | ABC7-42418200C9.1 | 0.45 |
| RN7SL237P | 0.58 | LRRC8C | 0.57 | RP11-126O22.5 | 0.54 | PTGIR | 0.52 | CTD-2015H6.2 | 0.5 | TNS1 | 0.48 | AP000476.1 | 0.46 | FMO2 | 0.45 |
| RP1-231P7P.1 | 0.58 | GLYATL1P2 | 0.57 | CYP4B1 | 0.54 | LINC01139 | 0.51 | SMOC2 | 0.5 | MEIS3P1 | 0.48 | IGFBP7 | 0.46 | ERG | 0.45 |
| RP11-63A23.2 | 0.58 | PODN | 0.57 | RP11-234O6.2 | 0.54 | VGLL2 | 0.51 | THBD | 0.5 | ZIM3 | 0.48 | LINC01037 | 0.46 | ADGRA2 | 0.45 |
| RP11-492A10.1 | 0.58 | RP11-544D21.2 | 0.56 | CTD-2536I1.2 | 0.54 | RP11-368M16.10 | 0.51 | RP11-126O22.8 | 0.5 | RP11-314D7.2 | 0.48 | CTD-2272D18.1 | 0.46 | DLGAP1 | 0.45 |
| RN7SKP218 | 0.58 | LRIT3 | 0.56 | HRASLS5 | 0.54 | RP11-242O24.3 | 0.51 | PRL | 0.5 | CCDC80 | 0.48 | CHRM3 | 0.46 | LDB2 | 0.45 |
| RP11-347L18.1 | 0.58 | NGFR | 0.56 | MYT1L | 0.53 | RP11-428F8.2 | 0.51 | RP11-570J4.2 | 0.49 | AC067961.1 | 0.47 | PTPRB | 0.46 | ZDHHC15 | 0.45 |
| AC013429.4 | 0.58 | OR2Z1 | 0.56 | NFASC | 0.53 | RP11-184J23.2 | 0.51 | HPRT1P1 | 0.49 | DDHD1 | 0.47 | RP11-455B3.1 | 0.46 | CNTN4 | 0.45 |
