# Supplementary material for: Comprehensive Analysis to Identify the Encoded Gens of Sodium Channels as a Prognostic Biomarker in Hepatocellular Carcinoma
Source: Front Genet. 2022 Jan 21;12:802067. doi: 10.3389/fgene.2021.802067 (PMC8815461; doi:10.3389/fgene.2021.802067)
Supplement: Supplementary file 5 [file DataSheet1.docx]

**Supplementary files online**

**1. Supplementary Table 1.** Univariate analyses of RFS and OS rates for 306 HCC patients after curative resection.

**2. Supplementary Table 2.** Correlation of SCN7A expression with clinicopathologic characteristics in 306 patients with HCC.

**3. Supplementary Table 3.** Cox multivariate analysis of prognostic factors to RFS and OS in 306 HCC patients after curative resection.

**1. Supplementary Table 1.** Univariate analyses of RFS and OS rates for 306 HCC patients after curative resection

| **Variables** | **Cases** | **RFS rate (%)** | | ***P* value** | **OS rate (%)** | | ***P* value** |
| --- | --- | --- | --- | --- | --- | --- | --- |
|  |  | **3 y** | **5 y** |  | **3 y** | **5 y** |  |
| Gender |  |  |  |  |  |  |  |
| Female | 36 | 82.3 | 72.1 |  | 79.3 | 71.5 |  |
| Male | 270 | 65.4 | 57.6 | 0.121 | 86.1 | 72.1 | 0.321 |
| Age (y) |  |  |  |  |  |  |  |
| ≤ 50 | 150 | 64.1 | 57.1 |  | 80.0 | 75.0 |  |
| > 50 | 156 | 70.5 | 61.2 | 0.283 | 80.1 | 68.7 | 0.397 |
| HbsAg |  |  |  |  |  |  |  |
| Negative | 42 | 74.5 | 68.7 |  | 78.6 | 70.5 |  |
| Positive | 264 | 66.3 | 57.7 | 0.147 | 80.3 | 72.0 | 0.944 |
| Child-Pugh classification**^l^** | |  |  |  |  |  |  |
| A | 302 | 67.7 | 59.4 |  | 80.5 | 72.0 |  |
| B | 4 | 50.0 | 50.0 | 0.079 | 50.0 | 50.0 | 0.166 |
| Alpha-fetoprotein (AFP, ng/ml) | | | | | | | |
| ≤ 20 | 135 | 65.4 | 56.9 |  | 87.4 | 81.3 |  |
| 20-400 | 69 | 71.0 | 63.1 |  | 73.9 | 61.7 |  |
| > 400 | 102 | 68.0 | 60.3 | 0.767 | 74.5 | 65.9 | **0.038** |
| Gamma glutamyl transferase (GGT, units/L) | | | | | | | |
| ≤ 50 | 184 | 75.7 | 66.3 |  | 82.6 | 79.2 |  |
| > 50 | 122 | 54.5 | 48.1 | **0.002** | 76.2 | 60.4 | **0.003** |
| Tumor size (cm) | |  |  |  |  |  |  |
| ≤ 5 | 161 | 74.9 | 65.3 |  | 85.1 | 78.0 |  |
| > 5 | 145 | 58.6 | 52.1 | **0.009** | 74.5 | 64.5 | 0.061 |
| Satellite nodule | |  |  |  |  |  |  |
| No | 264 | 71.9 | 63.8 |  | 83.3 | 75.0 |  |
| Yes | 42 | 36.0 | 21.0 | **< 0.001** | 59.5 | 50.4 | **< 0.001** |
| Tumor capsule | |  |  |  |  |  |  |
| No | 114 | 66.1 | 59.6 |  | 77.2 | 70.2 |  |
| Yes | 192 | 68.1 | 58.9 | 0.659 | 81.8 | 72.7 | 0.601 |
| Vascular invasion | |  |  |  |  |  |  |
| No | 252 | 69.1 | 59.8 |  | 82.9 | 74.7 |  |
| Yes | 54 | 58.8 | 58.8 | 0.326 | 66.7 | 56.7 | **0.004** |
| Ascites | |  |  |  |  |  |  |
| No | 284 | 68.3 | 60.0 |  | 80.3 | 72.0 |  |
| Yes | 22 | 53.9 | 48.5 | 0.379 | 77.3 | 67.5 | 0.481 |
| Tumor counts | |  |  |  |  |  |  |
| 1 | 251 | 70.5 | 63.2 |  | 83.7 | 75.2 |  |
| > 1 | 55 | 51.8 | 35.2 | **0.002** | 63.6 | 55.3 | **< 0.001** |
|  |  |  |  |  |  |  |  |
| (Continued) |  |  |  |  |  |  |  |
| **Variables** | **Cases** | **RFS rate (%)** | | ***P* value** | **OS rate (%)** | | ***P* value** |
|  |  | **3 y** | **5 y** |  | **3 y** | **5 y** |  |
| Adjacent organ invasion | |  |  |  |  |  |  |
| No | 257 | 68.3 | 60.2 |  | 82.1 | 74.7 |  |
| Yes | 49 | 62.9 | 53.8 | 0.485 | 69.4 | 55.6 | 0.061 |
| HCV-IgG | |  |  |  |  |  |  |
| No | 302 | 67.4 | 59.5 |  | 80.1 | 72.1 |  |
| Yes | 4 | 66.7 | 33.3 | 0.478 | 50.0 | 50.0 | **0.022** |
| Aspartate aminotransferase (AST, units/L) | | | | | | | |
| ≤ 40 | 198 | 72.9 | 65.2 |  | 80.8 | 76.2 |  |
| > 40 | 108 | 57.2 | 47.8 | **0.004** | 78.7 | 63.0 | 0.068 |
| Alanine aminotransferase (ALT, units/L) | | | | | | | |
| ≤ 40 | 184 | 73.5 | 67.3 |  | 83.7 | 76.9 |  |
| > 40 | 122 | 58.8 | 47.5 | **0.004** | 75.0 | 64.5 | **0.007** |
| Total bilirubin (umol/L) | |  |  |  |  |  |  |
| ≤ 17.1 | 217 | 68.8 | 61.0 |  | 79.7 | 71.4 |  |
| > 17.1 | 89 | 63.8 | 54.9 | 0.538 | 80.9 | 72.2 | 0.829 |
| Direct bilirubin (umol/L) | |  |  |  |  |  |  |
| ≤ 6.9 | 245 | 67.8 | 60.0 |  | 81.6 | 73.0 |  |
| > 6.9 | 61 | 65.1 | 55.6 | 0.736 | 73.8 | 66.5 | 0.188 |
| Albumin (g/L) | |  |  |  |  |  |  |
| ≤ 35 | 8 | 83.3 | 44.4 |  | 75.0 | 40.0 |  |
| > 35 | 298 | 66.9 | 59.4 | 0.938 | 80.2 | 72.6 | **0.045** |
| Tumor grade | |  |  |  |  |  |  |
| I / II | 230 | 71.3 | 61.1 |  | 83.5 | 75.0 |  |
| III / IV | 76 | 55.3 | 53.7 | 0.063 | 69.7 | 61.8 | **0.005** |
| Cirrhosis |  |  |  |  |  |  |  |
| No | 79 | 71.5 | 65.0 |  | 79.7 | 73.6 |  |
| Yes | 227 | 65.9 | 57.0 | 0.249 | 80.0 | 71.2 | 0.418 |
| TNM^2^ classification | |  |  |  |  |  |  |
| I / II | 241 | 72.4 | 64.6 |  | 79.7 | 73.6 |  |
| III / IV | 65 | 47.5 | 36.4 | **< 0.001** | 80.2 | 71.3 | **< 0.001** |
| SCN4A expression | |  |  |  |  |  |  |
| Negative | 128 | 69.9 | 60.8 |  | 79.7 | 73.1 |  |
| Positive | 178 | 65.6 | 57.9 | 0.333 | 80.3 | 70.8 | 0.903 |
| SCN7A expression | |  |  |  |  |  |  |
| Negative | 186 | 60.5 | 52.7 |  | 75.3 | 63.4 |  |
| Positive | 120 | 77.6 | 69.0 | **0.003** | 87.5 | 84.4 | **0.001** |

^1^There was no patient with Child–Pugh Class C disease.

^2^Tumor–node–metastasis (TNM) staging was evaluated based on the seventh edition of the American Joint Committee on Cancer (AJCC).

Abbreviations: DRD1, dopamine receptor D1. HCC, hepatocellular carcinoma. AFP, Alpha-fetoprotein. GGT, Gamma-glutamyl transferase. TNM, Tumor–Node–Metastasis.

**2. Supplementary Table 2.** Correlation of SCN7A expression with clinicopathologic characteristics in 306 patients with HCC**^1^**

| **Characteristics** | **Cases** | **SCN7A protein** | | |
| --- | --- | --- | --- | --- |
|  |  | **Low expression** | **High expression** | ***P* Value** |
| Gender |  |  |  |  |
| Female | 36 | 18 (50.0%) | 18 (50.0%) |  |
| Male | 270 | 168 (62.2%) | 102 (37.8%) | 0.158 |
| Age (y) |  |  |  |  |
| ≤ 50 | 150 | 45 (30.0%) | 105 (70.0%) |  |
| > 50 | 156 | 141 (90.4%) | 15 (9.6%) | < **0.001** |
| HbsAg |  |  |  |  |
| Negative | 42 | 29 (69.0%) | 13 (31.0%) |  |
| Positive | 264 | 157 (59.5%) | 107 (40.5%) | 0.238 |
| Child-Pugh classification**^l^** | |  |  |  |
| A | 302 | 183 (60.6%) | 119 (39.4%) |  |
| B | 4 | 3(75.0%) | 1 (25.0%) | 0.558 |
| Alpha-fetoprotein (AFP, ng/ml) | | | | |
| ≤ 20 | 135 | 92 (68.1%) | 43 (31.9%) |  |
| 20-400 | 69 | 37 (53.6%) | 32 (46.4%) |  |
| > 400 | 102 | 57 (55.9%) | 45 (44.1%) | 0.061 |
| Gamma glutamyl transferase (GGT, units/L) | | | | |
| ≤ 50 | 184 | 106 (57.6%) | 78 (42.4%) |  |
| > 50 | 122 | 80 (65.6%) | 42 (34.4%) | 0.162 |
| Tumor size (cm) |  |  |  |  |
| ≤ 5 | 161 | 84 (52.2%) | 77 (47.8%) |  |
| > 5 | 145 | 102 (70.3%) | 43 (29.7%) | **0.001** |
| Satellite nodule | |  |  |  |
| No | 264 | 154 (58.3%) | 110 (41.7%) |  |
| Yes | 42 | 32 (76.2%) | 10 (23.8%) | **0.028** |
| Tumor capsule |  |  |  |  |
| No | 114 | 75 (65.8%) | 39 (34.2%) |  |
| Yes | 192 | 111(57.8%) | 81 (42.2%) | 0.167 |
| Vascular invasion |  |  |  |  |
| No | 252 | 151 (59.9%) | 101 (40.1%) |  |
| Yes | 54 | 35 (64.8%) | 19 (35.2%) | 0.504 |
| Ascites |  |  |  |  |
| No | 284 | 174 (60.9%) | 111 (39.1%) |  |
| Yes | 22 | 13 (59.1%) | 9 (40.9%) | 0.866 |
| Tumor counts |  |  |  |  |
| 1 | 251 | 147 (58.6%) | 104 (41.4%) |  |
| > 1 | 55 | 39 (70.9%) | 16 (29.1%) | 0.089 |
|  |  |  |  |  |
| (Continued) |  |  | |  |
| **Characteristics** | **Cases** | **SCN7A protein** | | ***P* Value** |
|  |  | **Low expression** | **High expression** |  |
| Adjacent organ invasion | | | | |
| No | 257 | 149 (58.0%) | 108 (42.0%) |  |
| Yes | 49 | 37 (75.5%) | 12 (24.5%) | **0.021** |
| HCV-IgG |  |  |  |  |
| No | 302 | 182 (60.3%) | 120 (39.7%) |  |
| Yes | 4 | 4 (100.0%) | 0 (0%) | 0.106 |
| Aspartate aminotransferase (AST, units/L) | | | | |
| ≤ 40 | 198 | 113 (57.1%) | 85 (42.9%) |  |
| > 40 | 108 | 73 (67.6%) | 35 (32.4%) | 0.072 |
| Alanine aminotransferase (ALT, units/L) | | | | |
| ≤ 40 | 178 | 111 (62.4%) | 67 (37.6%) |  |
| > 40 | 128 | 75 (58.6%) | 53 (41.4%) | 0.506 |
| Total bilirubin (umol/L) | | | | |
| ≤ 17.1 | 217 | 139 (64.1%) | 78 (35.9%) |  |
| > 17.1 | 89 | 47 (52.8%) | 42 (47.2%) | 0.067 |
| Direct bilirubin (umol/L) | | | | |
| ≤ 6.9 | 245 | 151 (61.6%) | 94 (38.4%) |  |
| > 6.9 | 61 | 35 (57.4%) | 26 (42.6%) | 0.542 |
| Albumin (g/L) |  |  |  |  |
| ≤ 35 | 8 | 4 (50.0%) | 4 (50.0%) |  |
| > 35 | 298 | 182 (61.1%) | 116 (38.9%) | 0.527 |
| Tumor grade |  |  |  |  |
| I / II | 230 | 132 (57.4%) | 98 (42.6%) |  |
| III / IV | 76 | 54 (71.1%) | 22 (28.9%) | **0.034** |
| Cirrhosis |  |  |  |  |
| No | 79 | 48 (60.8%) | 31 (39.2%) |  |
| Yes | 227 | 138 (60.8%) | 89 (39.2%) | 0.996 |
| TNM classification | | | | |
| I / II | 241 | 127 (52.7%) | 114 (47.3%) |  |
| III / IV | 65 | 59 (90.8%) | 6 (9.2%) | **< 0.001** |

**^1^**Values of statistical significance are in bold.

**^2^**There is no patient with Child-Pugh Class C.

**3. Supplementary Table 3.** Cox multivariate analysis of prognostic factors to RFS and OS in 306 HCC patients after curative resection*****

| **Variables** | **β** | **SE** | **Hazard ratio**  **(95％CI)** | ***P* value** |
| --- | --- | --- | --- | --- |
| **RFS** |  |  |  |  |
| GGT | 0.289 | 0.202 | 1.335 (0.899~1.983) | 0.152 |
| Tumor size | 0.065 | 0.220 | 1.067 (0.693~1.641) | 0.769 |
| Satellite nodule | 1.082 | 0.505 | 2.951 (1.098~7.934) | **0.032** |
| Tumor counts | -0.344 | 0.472 | 0.709 (0.281~1.787) | 0.466 |
| AST | 0.138 | 0.230 | 1.148 (0.731~1.802) | 0.549 |
| ALT | 0.300 | 0.226 | 0.469 (0.299~0.735) | **0.001** |
| TNM classification | 0.176 | 0.278 | 1.192 (0.692~2.053) | 0.527 |
| SCN7A | -0.445 | 0.210 | 0.641 (0.425~0.967) | **0.034** |
| **OS** |  |  |  |  |
| AFP | 0.227 | 0.131 | 1.255 (0.971~1.622) | 0.083 |
| GGT | 0.399 | 0.227 | 1.490 (0.955~2.326) | 0.079 |
| Satellite nodule | -0.067 | 0.505 | 0.936 (0.348~2.516) | 0.895 |
| Vascular invasion | 0.168 | 0.286 | 1.182 (0.675~2.072) | 0.558 |
| Tumor counts | 0.537 | 0.450 | 1.711 (0.708~4.133) | 0.233 |
| HCV-IgG | 1.105 | 0.619 | 3.018 (0.896~10.159) | 0.075 |
| ALT | 0.276 | 0.230 | 1.318 (0.839~2.069) | 0.231 |
| Albumin | -1.146 | 0.494 | 0.318 (0.121~0.837) | **0.020** |
| Tumor grade | -0.041 | 0.252 | 0.960 (0.587~1.572) | 0.872 |
| TNM classification | 0.636 | 0.291 | 1.890 (1.068~3.345) | **0.029** |
| SCN7A | -0.595 | 0.266 | 0.552 (0.328~0.929) | **0.025** |

*Only the factors that were significantly affect RFS or OS are showed in this table.
